# Compositions of gut microbiota before and shortly after hepatitis C viral eradication by direct antiviral agents

**DOI:** 10.1038/s41598-022-09534-w

**Published:** 2022-03-31

**Authors:** Yao-Chun Hsu, Chih-Cheng Chen, Wei-Hsiang Lee, Chi-Yang Chang, Fu-Jen Lee, Cheng-Hao Tseng, Tzu-Haw Chen, Hsiu J. Ho, Jaw-Town Lin, Chun-Ying Wu

**Affiliations:** 1 Division of Gastroenterology and Hepatology, E-Da Hospital, Kaohsiung, Taiwan; 2 School of Medicine, I-Shou University, Kaohsiung, Taiwan; 3 Institute of Biomedical Informatics, National Yang Ming Chiao Tung University, Taipei, Taiwan; 4 Division of Gastroenterology and Hepatology, Fu-Jen Catholic University Hospital, Fu-Jen Catholic University, New Taipei, Taiwan; 5 Division of Gastroenterology and Hepatology, E-Da Cancer Hospital, Kaohsiung, Taiwan; 6 Division of Translational Research, Taipei Veterans General Hospital, No.201, Sec. 2, Shipai Rd., Beitou District, Taipei City, 11217 Taiwan; 7 Department of Public Health, China Medical University, Taichung, Taiwan; 8 Taiwan Microbiota Consortium, Taipei, Taiwan; 9 College of Medicine, National Yang Ming Chiao Tung University, Taipei, Taiwan

**Keywords:** Bacteriology, Gastroenterology, Hepatitis C

## Abstract

It is unclear whether dysbiosis in hepatitis C virus (HCV) infected patients results from the viral infection per se or develops as a result of hepatic dysfunction. We aimed to characterize compositions in gut microbiome before and shortly after HCV clearance. In this prospective cohort study, adult patients with confirmed HCV viremia were screened before receiving direct antiviral agents. Those with recent exposure to antibiotics or probiotics (within one month), prior abdominal surgery, or any malignancy were ineligible. Stool was collected before antiviral therapy started and at 12 weeks after the treatment completed. From the extracted bacterial DNA, 16 s rRNA gene was amplified and sequenced. Each patient was matched 1:2 in age and sex with uninfected controls. A total of 126 individuals were enrolled into analysis. The gut microbiome was significantly different between HCV-infected patients (*n* = 42), with or without cirrhosis, and their age-and sex-matched controls (*n* = 84) from the levels of phylum to amplicon sequence variant (all *p* values < 0.01 by principal coordinates analysis). All patients achieved viral eradication and exhibited no significant changes in the overall composition of gut microbiome following viral eradication (all *p* values > 0.5), also without significant difference in alpha diversity (all *p* values > 0.5). For the purpose of exploration, we also reported bacteria found differently abundant before and after HCV eradication, including *Coriobacteriaceae*, *Peptostreptococcaceae*, *Staphylococcaceae, Morganellaceae, Pasteurellaceae*, *Succinivibrionaceae,* and *Moraxellaceae*. Gut microbiota is altered in HCV-infected patients as compared with uninfected controls, but the overall microbial compositions do not significantly change shortly after HCV eradication.

## Introduction

Alterations of the gut microbiome are frequently observed in patients with chronic liver diseases of various etiologies including hepatitis C virus (HCV) infection^1–3^. Severity of the dysbiosis is correlated with stages of the disease and has been implicated in the pathogenesis of disease progression^4,5^. Moreover, preliminary data suggest that microbial compositions may help stratify the risk of clinical outcomes^6,7^. However, the associations among the etiology of the liver disease, composition of the gut microbiome, and disease progression are likely to be complex with the causal relationship difficult to be determined^8^. It has not been clarified whether HCV infection, in and of itself, causes alterations of the gut microbiome that drive disease progression^5,9,10^, or alternatively, whether gut dysbiosis results from longstanding inflammation of the liver and/or resultant hepatic dysfunction^11–13^, instead of the viral infection per se.

Ideally, gut microbiota collected around acute HCV infection should be measured to determine if acquisition of the viral infection per se leads to microbial alterations. Such an approach requires collection of microbial data prior to occurrence of the infection and is understandably very difficult. Alternatively, measuring the changes in gut microbiome right after viral eradication provides an opportunity to answer this issue. This approach is particularly appealing when direct antiviral agents (DAAs) became routinely available to eradicate HCV infection in nearly all infected patients with negligible side effects within 2 or 3 months^14,15^. Because the DAA regiments are specific against the viral proteins, highly effective, easily tolerable, and short in duration^16^, the concerns that microbial communities could be influenced by off-target pharmacological effects, dietary adjustments, or behavioral changes over a prolonged period of time may be mitigated. Nevertheless, current literature is not only sparse but also limited by pitfalls such as exclusive enrollment of patients with cirrhosis, delayed measurement of microbiome, and small numbers of participants.

In this prospective cohort study of patients chronically infected with HCV, we measured and analyzed microbial compositions from fecal samples collected prior to the start of all-oral DAA regimens and at 12 weeks after completion of the antiviral treatment, in order to clarify the changes in gut microbiome along with the predicted functional profiles shortly following eradication of HCV infection.

## Methods and materials

### Study design and participants

This is a prospective cohort study conducted at three teaching hospitals in Taiwan (i.e., E-Da Hospital, E-Da Cancer Hospital, and Fu-Jen Catholic University Hospital). From March 09, 2018 to March 06, 2019, adult patients with chronic HCV infection were screened for eligibility if they were aged 20 years or above, presenting with active viremia confirmed by detectable viral RNA in the serum, and about to receive interferon-free all-oral DAA regimens that were approved by the regulatory agency in Taiwan for the treatment of HCV infection. They were ineligible for any of the following conditions: interferon included in the antiviral regimen, recent treatment with antibiotics, steroid, immunosuppressant, or cytotoxic agents within 3 months, history of malignancy or organ transplantation, and coinfection with human immunodeficiency virus. This study was conducted in accordance with the principles of the Declaration of Helsinki and the protocol (EMRP-107–006 and EMRP-107–097) was reviewed and approved by the ethics committee at each site (Institutional Review Board of the E-Da Hospital, Fu Jen Catholic University Institutional Review Board, and Taichung Veterans General Hospital Institutional Review Board). All participants provided written informed consent.

We also included individuals without HCV infection by retrieving data from persons who voluntarily donated their stool for microbial analysis in a health checkup program at the Taichung Veterans General Hospital (Taichung, Taiwan) from January 2016 to January 2018. These healthy volunteers and the comprehensive examinations they received have been characterized in details^17^. The stool was collected, stored, processed, and analyzed according to the protocols exactly the same as HCV-infected patients in this study. All the HCV-uninfected controls were confirmed serologically by absence of anti-HCV antibody and were matched 2:1 with HCV-infected patients in age and biological sex.

### Collection of clinical data and stool sample

At baseline, enrolled patients were interviewed with structured questionnaire and examined with abdominal sonography as well as physically. They also received blood tests for cell counts, biochemistry, viral serology, and HCV RNA prior to taking the antiviral medications. In the absence of pathological proof, presence of cirrhosis was clinically diagnosed mainly by the typical features on sonography including uneven surface, coarse echotexture, and splenomegaly^18^. During the antiviral treatment, patients were followed at a maximal interval of 4 weeks to ensure adherence to the therapy. The attainment of sustained virological response at 12 weeks (i.e. HCV RNA undetected in serum at 12 weeks after completion of the antiviral therapy) defined successful viral eradication^14^. Blood examinations were carried out at the local laboratory in each hospital. The lower bound for HCV RNA to be detectable at serum was 15 IU/mL in all three hospitals.

Stool was collected within 24 h before commencement of the antiviral treatment and also at 12 weeks after completion of the therapy using a standardized kit^1^. The process of stool collection was previously described^19^. In brief, patients were instructed to defecate stool directly into the collection kit and mail the kit along with a cooler bag to the laboratory. The collected specimens were stored at −20 °C before further processing.

### Extraction, amplification, and sequencing of bacterial DNA

DNA was extracted from the stool specimens, which were kept on ice, with Qiagen DNA isolation kit (Qiagen, MD, USA). In general, 15–20 μg DNA was extracted from 200 mg of stool samples. The concentration and quality of isolated DNA were confirmed by NanoDrop ND-1000 (Thermo, DE, USA) with the following standards: a minimum of 500 ng, 260/280 ratio of 1.7–1.8, and 260/230 ratio of 1.8–2.2.

The hypervariable V3-V4 regions of bacterial 16S rRNA genes were amplified by polymerase chain reaction (PCR) with the bar-coded universal primers: 341F (Forward; 5-CCTACgggNggCWgCAg-3) and 805R (Reverse; 5-gACTACHCgggTATCTAATCC-3). Library construction and subsequent sequencing of the amplified DNAs were performed by Genomics BioScience (Taipei, Taiwan). A pair-end library was constructed with an insert size of 465 base pairs for each sample per the manufacturer’s instructions (Illumina, Wilmington, DE, USA). High-throughput sequencing was performed on the Illumina MiSeq 2000 platform (Illumina, Wilmington, DE, USA).

Amplicons of the 16S rRNA genes were analyzed by the bioinformatics experts at the Germark Biotechnology (Taichung, Taiwan). In brief, forward and reverse reads were filtered and trimmed, inferenced then merged with 10 base pairs as the minimal overlap. Merged reads were constructed to an amplicon sequence variant (ASV) table, a higher-resolution version of the operational taxonomic unit (OTU) table. Chimeric reads were also removed, and remaining sequences were aligned to Silva database (version 138) for taxonomic assignments. All steps above were done by DADA2^20^.

### Statistical analysis

Diversity indices (Chao1, Shannon, Simpson, and inverted Simpson) were calculated using R software with the package of phyloseq^21^. In addition, cladogram and linear discriminant analysis (LDA) by LEfSe were carried out for high-dimensional class comparisons^22^.

The gut metabolic modules (GMMs) are bacterial and archaeal metabolic pathways associated with human gut, and mainly focused on anaerobic fermentation processes^23^. Each module is composed of a group of prokaryotic and archaeal KEGG Orthology (KO)^24–26^ to describe an enzymatic process converting input compound to output metabolite. GMMs were firstly grouped by their position in the gut metabolic map (i.e., input, central, and output, of which the module number was 75, 11, and 17, respectively), then by 10 metabolic categories and 30 subcategories. Currently, there are 103 GMMs in total. To infer the GMMs profile, PICRUSt2 was used to predict the KO profile by analyzing the ASV-level abundance data of microbiota. Following that, the Omixer-RPM7 (https://github.com/raeslab/omixer-rpm) was used for GMM prediction by subjecting KO profile as input with the setting of coverage as -1 (i.e., the pathway KO coverage threshold was learned from the coverage distribution of all modules). To identify GMMs with differential abundances, enrichment analysis was performed by a two-group comparison for medians using two-tailed Wilcoxon test with a Benjamini–Hochberg false discovery rate correction to adjust q-values for multiple testing. In addition, the association analysis mentioned above was performed accordingly to assess its overall association with variables of interest.

## Results

### Characteristics of the study cohorts

A total of 126 individuals were included into analysis, among whom there were 42 patients with HCV infection and 84 uninfected controls matched by age and sex (Table 1). HCV-infected patients were characterized by higher serum levels of AST, ALT, and creatinine, a lower platelet count, and a higher proportion with liver cirrhosis, although the two groups were similar in body mass index and prevalence of hepatitis B virus (HBV) infection. Serum HBV DNA was undetectable in most patients with dual infection of HBV and HCV (Supplementary Table 1). Two dually infected patients also received antiviral therapy for HBV during the treatment for HCV.

**Table 1:** Clinical characteristics of the study population.

Characteristics	Healthy controls (*n* = 84)	Chronic hepatitis C patients (*n* = 42)	P**
Age, year	58.0 (53.0–64.0)	59.0 (53.2–67.8)	0.25*
Male gender, *n* (%)	36 (42.9%)	17 (40.5%)	0.95*
Body mass index, Kg/m^2^	24.2 (21.8–26.4)	24.8 (23.1–26.4)	0.26*
AST, U/L	25.0 (20.5–29.5)	46.5 (27.0–91.5)	< .001*
ALT, U/L	28.0 (19.2–38.5)	50.0 (30.5–106.5)	< .001*
Albumin, g/dL	4.5 (4.3–4.6)	4.4 (4.2–4.6)	0.067*
Creatinine, mg/dL	0.8 (0.6–0.9)	0.9 (0.8–1.1)	< .001*
Platelet, 10^3^/µL	249.0 (203.5–294.5)	191.0 (158.0–241.0)	< .001*
Chronic hepatitis B, n (%)	10 (11.9%)	8 (19.0%)	0.45*
Cirrhosis, n (%)	0	6 (14.3%)	< .001*
Hepatitis C viral load, IU/mL	Not applicable	835,500 (336,256–3,434,634)	
**Hepatitis C genotype**			
G1a	Not applicable	2 (4.8%)	
G1b	Not applicable	18 (42.8%)	
G2	Not applicable	20 (47.6%)	
G6	Not applicable	2 (4.8%)	
**Antiviral regimens**			
Sofosbuvir plus Ledipasvir	Not applicable	26 (61.9%)	
Elbasvir plus Grazoprevir	Not applicable	8 (19.0%)	
Sofosbuvir plus Ribavirin	Not applicable	6 (14.3%)	
Others	Not applicable	2 (4.8%)	
**Measured at 12 weeks off therapy**			
AST, U/L	Not applicable	24.0 (20.0–32.0)	< .001^+^
ALT, U/L	Not applicable	17.0 (14.0–24.0)	< .001^+^
Platelet, 10^3/^µL	Not applicable	195 (148–240)	0.26^+^

Data are expressed as median (interquartile range) or number (percentage).

Abbreviations: *ALT* alanine aminotransferase, *AST* aspartate aminotransferase.

**Mann–Whitney U test for continuous variables and Fisher’s exact test for categorical variable; *indicated comparison between healthy controls and patients with chronic hepatitis C; ^+^indicated paired comparison for measurements before and 12 weeks after eradication of hepatitis C virus infection.

**Figure 1:**
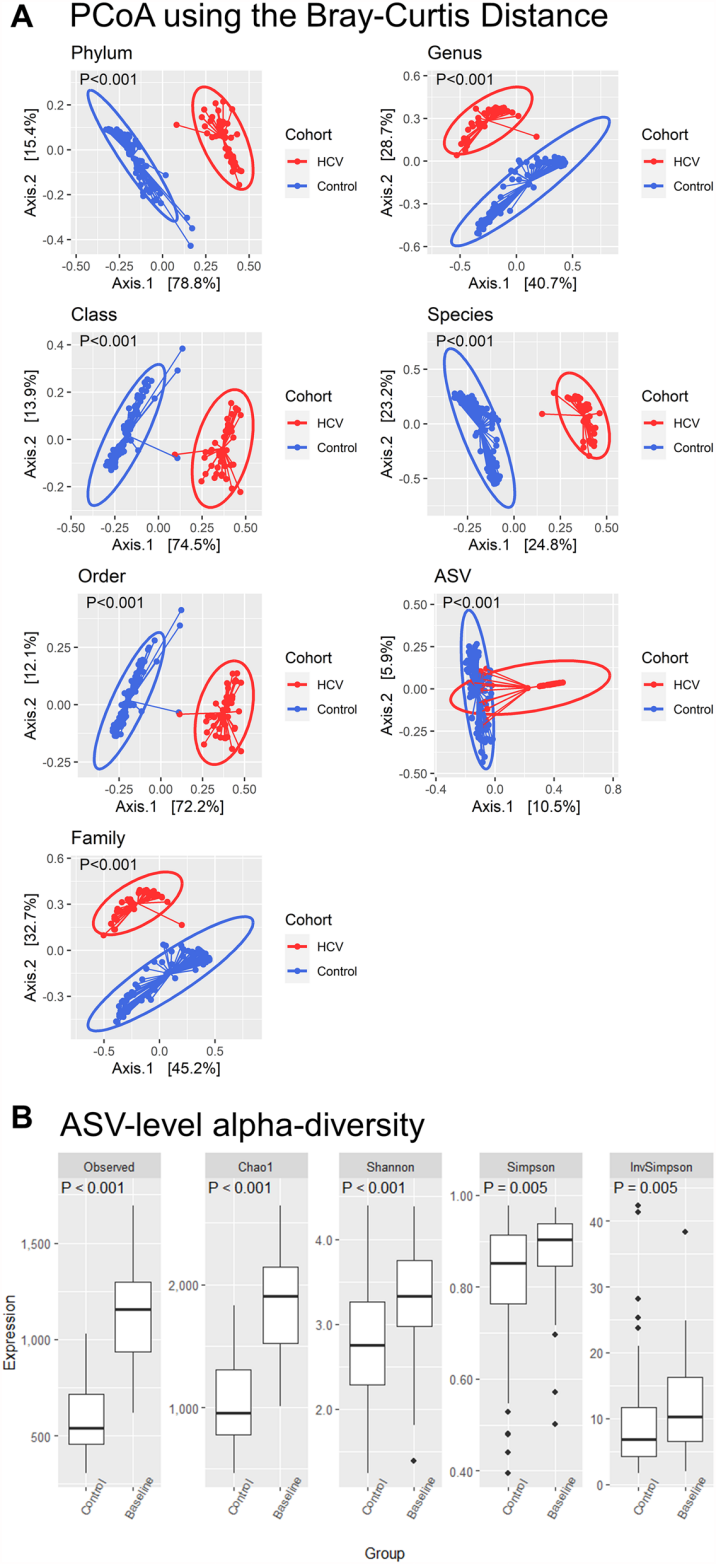
Principal coordinate analyses for the gut microbiome from phylum to amplicon sequence variant between patients chronically infected with hepatitis C virus and uninfected controls (**A**). Metrics of alpha diversity for gut microbiome in uninfected control and hepatitis C patients (**B**).

Compositions of the intestinal microbiome were significantly different between HCV-infected patients and uninfected controls, from the taxonomic levels of phylum to ASV (Fig. 1A). The two groups still differed in principal components of microbiome after patients with cirrhosis were excluded from analysis as a sensitivity test (Supplementary Fig. 1). Not surprisingly, the alpha-diversity of gut microbiome, as measured by observed species, Chao1 index, Shannon index, Simpson index, and inverted Simpson index, were significantly different between uninfected control and hepatitis C patients (Fig. 1B).

### Principle components of overall gut microbiome before and after eradication of HCV

**Figure 2:**
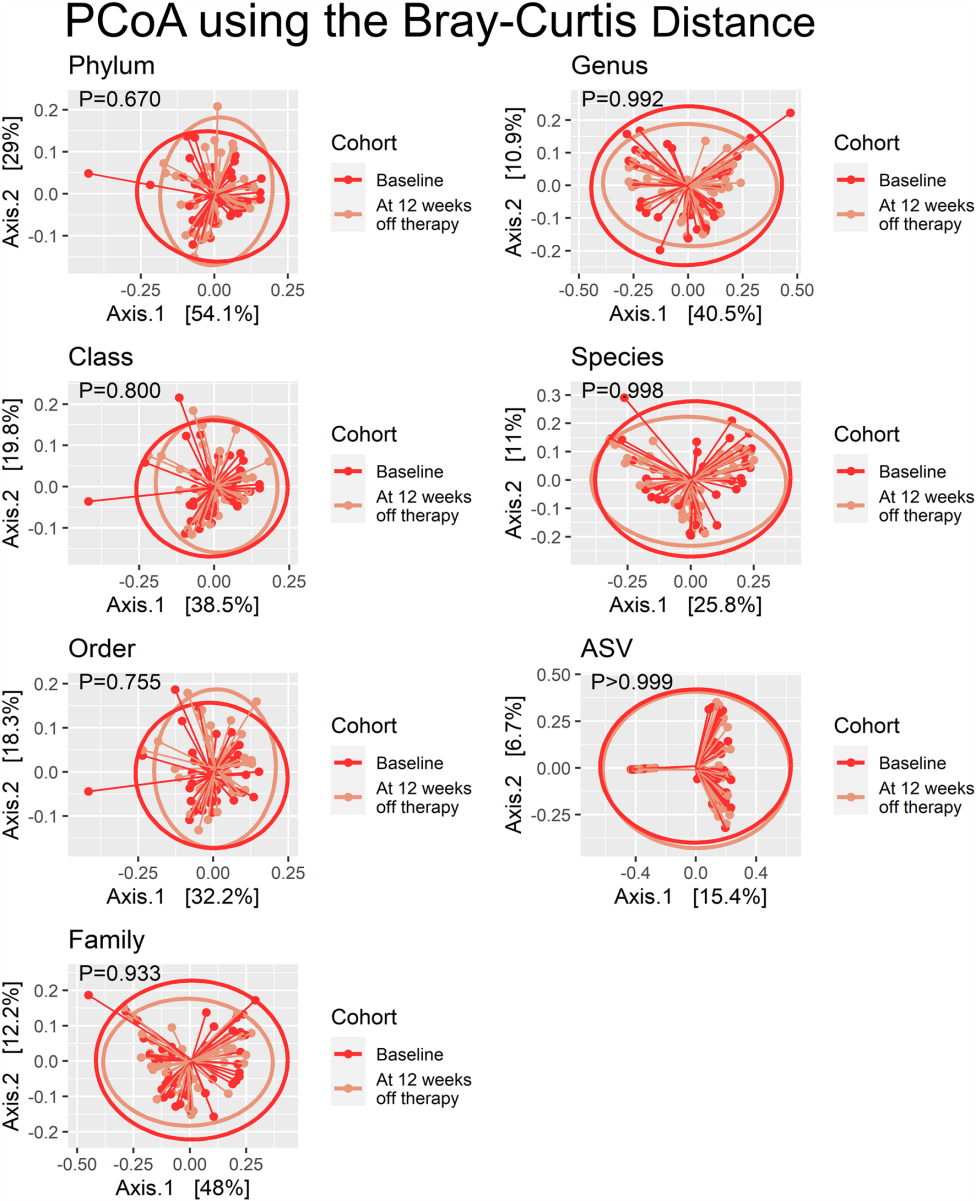
Principal coordinate analyses for the microbial compositions measured before and shortly after (12 weeks) eradication of hepatitis C virus infection.

**Figure 3:**
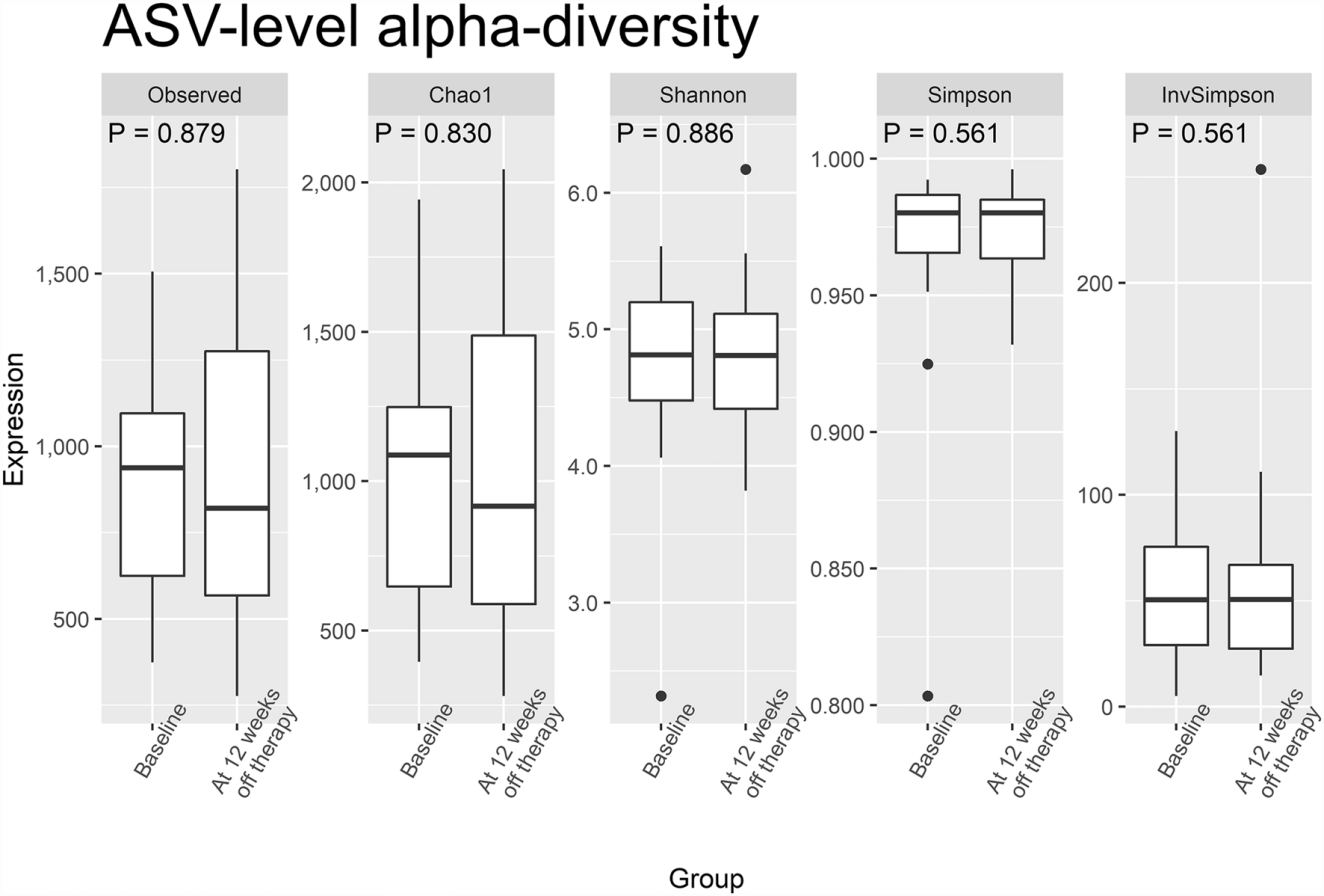
Metrics of alpha diversity for gut microbiome at baseline and 12 weeks following eradication of hepatitis C virus infection.

All HCV-infected patients successfully eradicated the virus with absence of viremia documented at the end of antiviral therapy and confirmed at 12 weeks following treatment cessation. Besides, there was no significant change in HBV viremia in patients with dual infection. As depicted by the PCoA (Fig. 2), the overall compositions of gut microbiomes measured at the pretreatment baseline and at 12 weeks after viral eradication were not significantly different from the levels of phylum to ASV. Similarly, the alpha-diversity of gut microbiome, as measured by observed species, Chao1 index, Shannon index, Simpson index, and inverted Simpson index, were not significantly different from baseline to 12 weeks after the viral infection was eradicated (Fig. 3).

### Exploration for changes in relative abundance of microbiomes after HCV clearance

**Figure 4:**
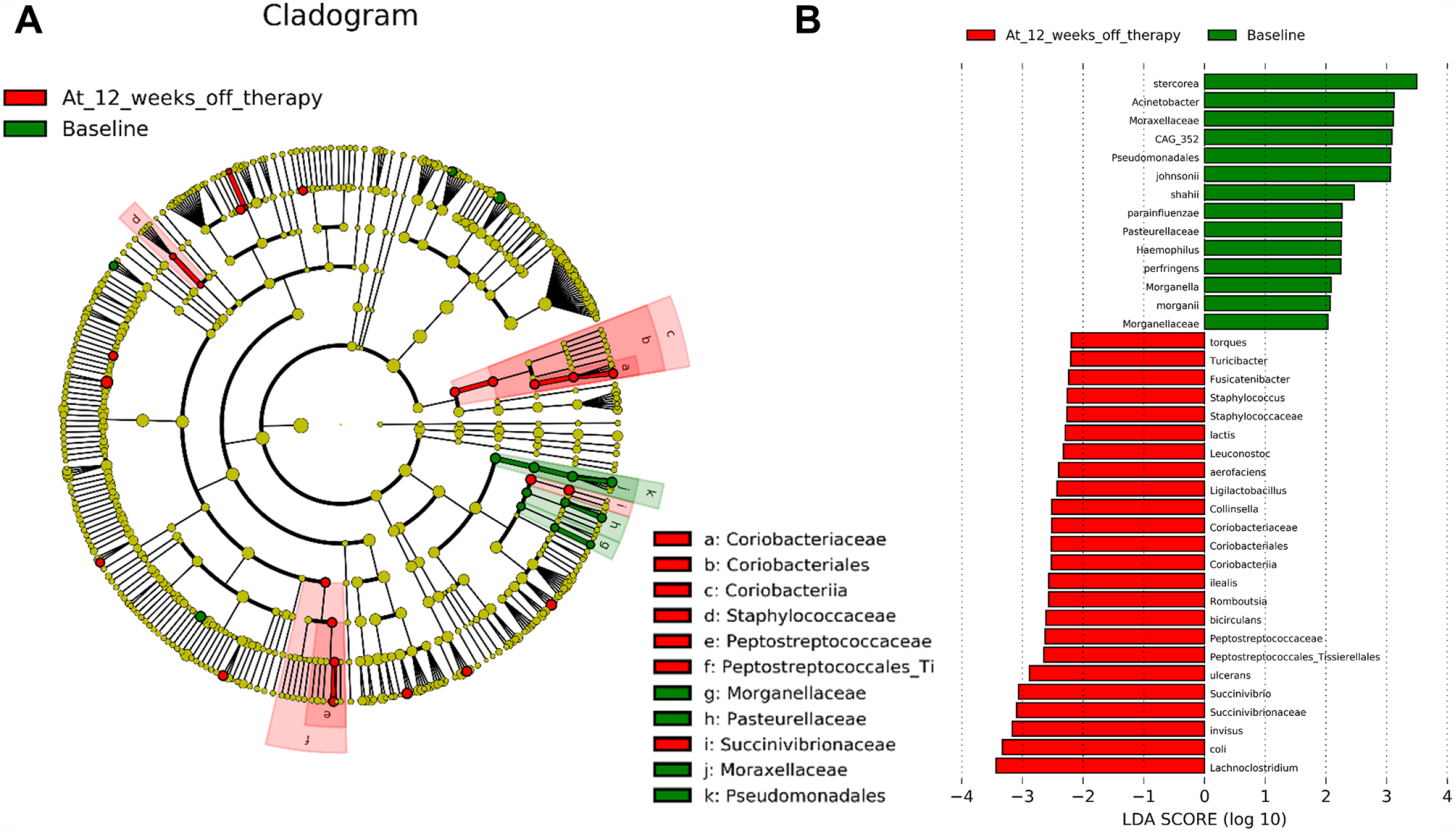
Linear discriminant analysis effect size (LEfSe) and linear discriminant analysis (LDA) to differentiate between gut microbiome measured before and 12 weeks after eradication of hepatitis C virus (HCV). In the left panel (**A**), cladogram showing the most discriminative bacteria identified by LEfSe. Red or green regions/branches indicate increase or decrease in the relative abundance of bacterial population following HCV clearance. In the right panel (**B**), LDA scores indicate significant differences in the microbial compositions before and after eradication of HCV infection.

The discriminant analysis uncovered distinct features of the gut microbiota before and after eradication of HCV infection (Fig. 4). As compared with the pretreatment status, the gut microbiome measured at 12 weeks off treatment were enriched in *Coriobacteriaceae*, *Staphylococcaceae*, *Peptostreptococcaceae*, and *Succinivibrionaceae*. On the other hand, the relative abundance decreased after viral clearance in *Morganellaceae, Pasteurellaceae, and Moraxellaceae*.

### Exploration of metagenomic functions predicted from the microbial changes

**Figure 5:**
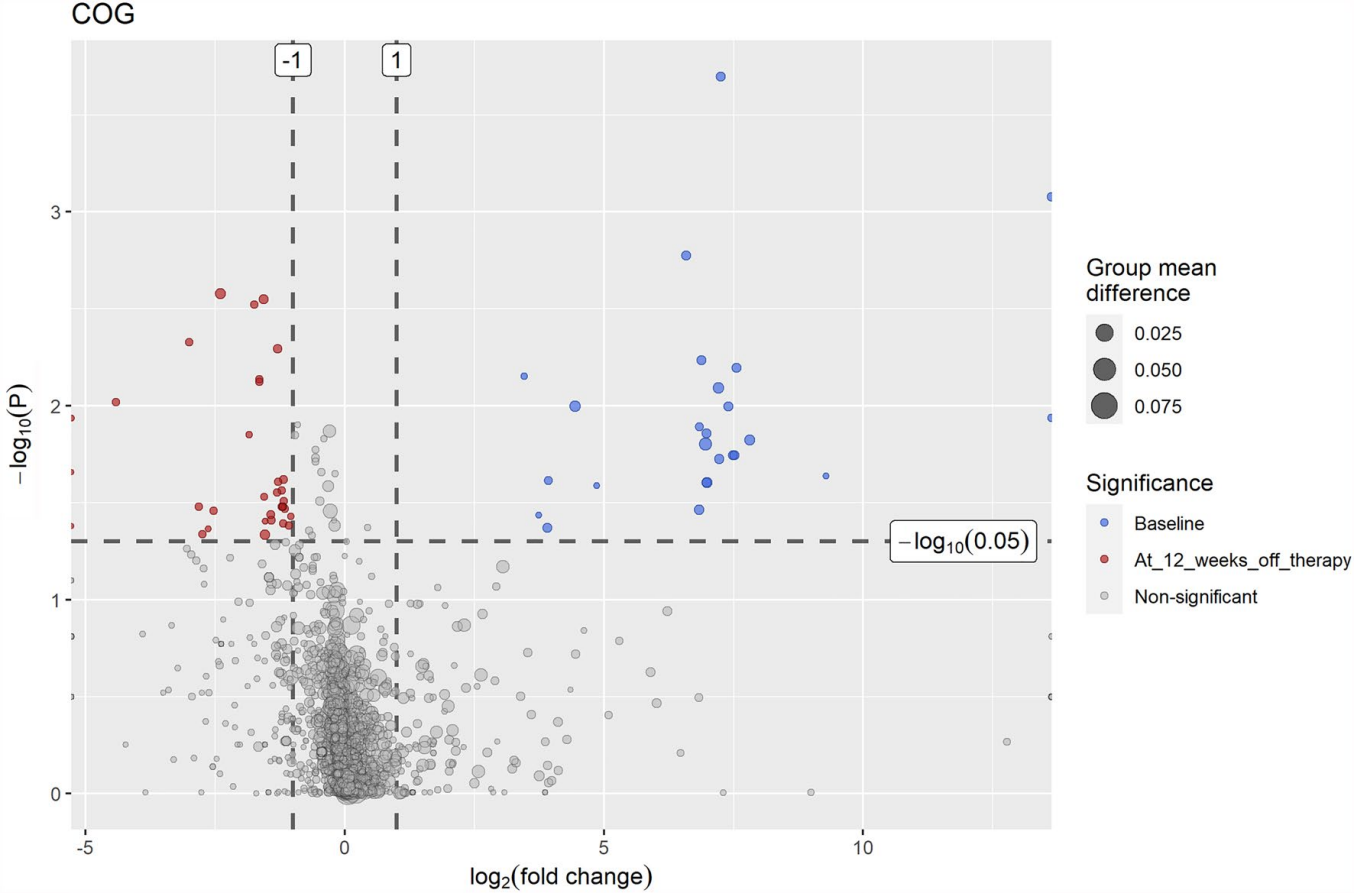
Volcano plot showing differences in the metabolic functional profiles of the gut microbiome, as predicted by the clusters of orthologous groups (COGs) of proteins from the microbial genes, between baseline status and 12 weeks after eradication of hepatitis C virus infection. The colored (red or blue) regions indicate statistically significant (*p* < 0.05) differences with a magnitude of at least one-fold.

Statistically significant (*P* < 0.05) changes with a magnitude of at least one folds in the metabolic functional profiles that were predicted from the abundance of clusters of orthologous groups (COGs) of proteins in gut microbiome from baseline to 12 weeks post viral clearance were illustrated by a volcano plot (Fig. 5). For instance, the predicted metabolic profiles after viral eradication were enriched in Tagatose-1,6-bisphosphate aldolase (COG 3684, *P* = 0.005) and a predicted Uncharacterized protein with an alpha/beta hydrolase fold (COG 4814, *P* = 0.003), whereas the abundance of Spo0M, a sporulation-control protein (COG 4326, *P* < 0.001), was predicted to be reduced after eradication of the virus. Details of the functional predictions were provided in the supplement (Supplementary Table 2).

## Discussion

This prospective cohort study demonstrated that gut microbiome in patients chronically infected with HCV, with or without liver cirrhosis, was different from that in uninfected individuals. In HCV-infected patients who received DAA treatment, however, there were no significant changes in the measurements of alpha diversities and principal components of the gut microbiome measured at 12 weeks following eradication of the virus. For the purpose of exploration, we found gut microbiome was enriched in *Coriobacteriaceae*, *Staphylococcaceae*, *Peptostreptococcaceae*, and *Succinivibrionaceae,* while less representative in *Morganellaceae, Pasteurellaceae, and Moraxellaceae* after viral eradication. While reaffirming that gut microbiome was different in patients infected with HCV as compared to uninfected individuals, our findings further suggested that HCV infection per se did not acutely (within the timeframe of 12 weeks) alter the profile of microbial composition. These data also reassured that DAA treatment did not exert substantiative effects on the intestinal commensals. The microbial differences as shown in the discriminative analyses were explorative in essence, requiring validation and comparison by further research.

Intestinal dysbiosis is frequently observed in patients with various chronic liver diseases, particularly in those with advanced liver fibrosis or cirrhosis^8,27,28^, but the causation remains largely elusive. One hypothesis is that the etiology of a liver disease, such as viral infection, alcohol exposure, or metabolic derangement is able to alter the gut microbiota by itself. In turn, the microbial alterations may exacerbate hepatic inflammation and fibrosis through endotoxemia in the portal circulation^27^. Alternatively, the etiological factor is not, in and of itself, the cause of dysbiosis. Instead, structural and physiological derangements that result from long-standing hepatic dysfunction (e.g., increased intestinal permeability, splanchnic vasodilation, or impaired antimicrobial immunity) are the driving force of the dysbiosis and consequentially the vicious cycle^8^. Oh and colleagues recently reported that a core signature of the gut microbiome could accurately identify patients with liver cirrhosis across geographically separated cohorts, regardless of the different etiologies, genetic makeups, and environmental exposures among individuals^29^. In view of the universal nature of this microbial signature, a common pathophysiological mechanism rather than an etiology-specific one is more plausible to account for the gut dysbiosis in various liver diseases at an advanced stage.

Existent literature pertaining the effects of HCV eradication on gut microbiota was very scanty and limited. In an earlier study involving 105 patients with HCV-related cirrhosis, Bajaj and colleagues reported that gut dysbiosis did not differ between patients who had previously eradicated the virus and those who remained viremic^13^. In agreement with our study, their findings suggested that HCV viremia alone was not associated with a distinct pattern of microflora when there were no differences in demographic, comorbidity, cirrhosis severity, or medication. This study, however, was limited by the use of obsolete regimens based on interferons and the lack of serial measurements in a same person. Recently, Perez-Matute and colleagues found the overall gut microbiota did not change following DAA treatment in 22 patients without cirrhosis^12^. There were no significant differences in the overall microbial compositions measured before the antiviral treatment, at completion of the treatment, and 3 months after completion of the treatment. These results, consistent with ours, demonstrated that neither exposure to DAAs nor viral clearance (at least within 3 months) would reshape the gut microbiota. Nevertheless, the study by Perez-Matute and colleagues was limited by a smaller sample size and restricted eligibility to a milder status of the disease severity.

Our findings appeared to contradict the study reported by Ponziani and colleagues, in which alpha diversity significantly improved along with a shift in the overall gut microbial composition following HCV eradication in 12 patients treated with DAA regimens^11^. The discrepant results require careful interpretation. The gut microbiota was not measured until one year after antiviral therapy in the study by Ponziani and colleagues, in contrast to our study and that by Perez-Matute et al., in which the measurement was carried out shortly after HCV eradication. Because cure of HCV infection effectively ameliorates hepatitis, prevents histological deterioration, and restores hepatic function over time, the significant microbial changes observed by Ponziani et al., may result from pathophysiological improvements that accrue from HCV eradication rather than indicated an etiology-specific change that corresponds to presence or absence of the viral infection. Besides, their study contained only 12 patients and the liver disease had progressed to cirrhosis in all of them. Accordingly, caution is warranted before generalizing their findings.

We acknowledge the following limitations. First, treatment was not randomized and thus the effects on gut microbiota could have been confounded. Nonetheless, it could not be ethical to randomly assign HCV-infected patients not to receive antiviral therapy. Second, we agreed that the sample size might be insufficient to uncover subtle differences that required a larger statistical power to be distinguished. Despite this limitation, our study was relatively large as compared with prior studies and could add to our understanding in this field with novel findings. Third, our study focused on short-term changes and outcomes. A longer period of follow-up is essential to evaluate whether recovery of hepatic dysfunction improved gut dysbiosis associated with HCV infection. Finally, caution is warranted before generalizing our findings to the diverse populations of HCV-infected patients. For instance, our study was not specifically designed for patients with HBV- and HCV- dual infection although HBV viremia was generally low and likely inconsequential in our co-infected patients. Further dedicated research was needed to address whether this special population was unique.

In conclusion, this prospective cohort study confirmed the alterations of gut microbiota in HCV-infected patients as compared with uninfected controls, and found no significant changes in the overall microbial compositions shortly after eradication of the virus by DAA treatment. Our data suggest that presence or absence of HCV infection per se does not reshape the profile of gut microflora. Besides, we reported bacteria that were found differently abundant before and after HCV eradication in our explorative analysis. Our findings provide important clues to elucidate the complex association of chronic hepatitis C with gut dysbiosis and may inspire future research for external validation and further exploration.

## Supplementary Information

Supplementary Legends.

Supplementary Information 2.

Supplementary Information 3.

Supplementary Information 4.
