# Supplementary material for: Compositions of gut microbiota before and shortly after hepatitis C viral eradication by direct antiviral agents
Source: Sci Rep. 2022 Mar 31;12:5481. doi: 10.1038/s41598-022-09534-w (PMC8971444; doi:10.1038/s41598-022-09534-w)
Supplement: Supplementary file 1 — Supplementary Legends. [file 41598_2022_9534_MOESM1_ESM.docx]

**SUPPLEMENTARY table**

**Supplementary Table 1.** Serum HBV DNA during antiviral therapy for HCV in patients with dual infection

**Supplementary Table 2.** Changes in functional predictions following eradication of hepatitis C virus infection

**SUPPLEMENTARY Figure Legends**

**Supplementary Figure 1.** Principal coordinate analyses for the gut microbiome from phylum to amplicon sequence variant between patients chronically infected with hepatitis C virus and uninfected controls (excluded cirrhotic patients).
