# Supplementary figures and images for: Compositions of gut microbiota before and shortly after hepatitis C viral eradication by direct antiviral agents
Source: Sci Rep. 2022 Mar 31;12:5481. doi: 10.1038/s41598-022-09534-w (PMC8971444; doi:10.1038/s41598-022-09534-w)

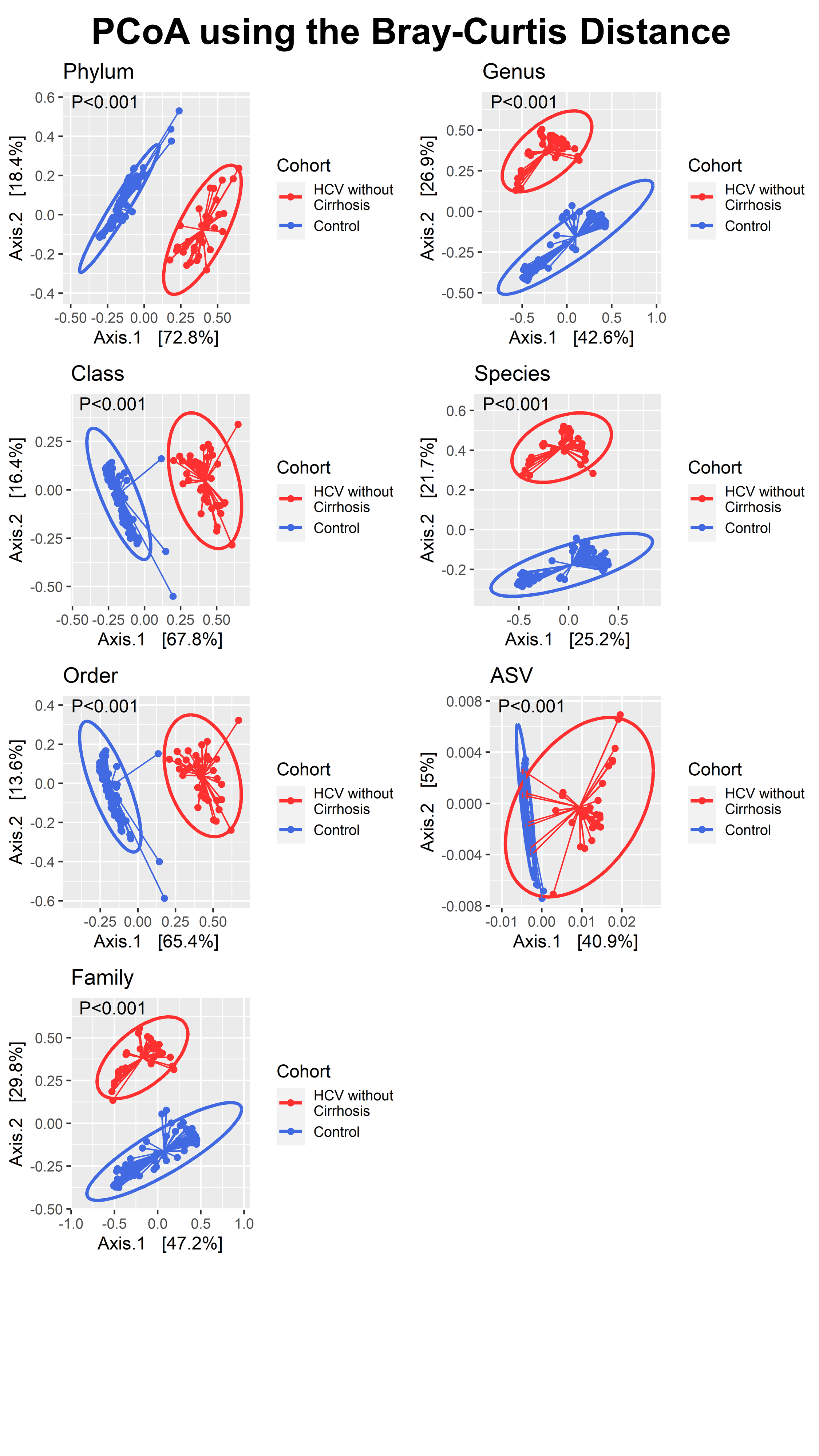

Supplement: Supplementary file 2 — Supplementary Information 2. [file 41598_2022_9534_MOESM2_ESM.tif]
