# Supplementary material for: Compositions of gut microbiota before and shortly after hepatitis C viral eradication by direct antiviral agents
Source: Sci Rep. 2022 Mar 31;12:5481. doi: 10.1038/s41598-022-09534-w (PMC8971444; doi:10.1038/s41598-022-09534-w)
Supplement: Supplementary file 3 — Supplementary Information 3. [file 41598_2022_9534_MOESM3_ESM.docx]

**Supplementary Table 1.** Serum HBV DNA during antiviral therapy for HCV in patients with dual infection

| Case number | Age | Sex | HBV DNA, baseline | HBV DNA, SVR12 |
| --- | --- | --- | --- | --- |
| 1* | 78 years | Male | Undetectable | Not available |
| 2* | 52 years | Male | 3470 IU/mL | Undetectable |
| 3 | 72 years | Male | Undetectable | Undetectable |
| 4 | 45 years | Female | Undetectable | Undetectable |
| 5 | 53 years | Female | Not available | Not available |
| 6 | 66 years | Female | Undetectable | Undetectable |
| 7 | 51 years | Female | Not available | Not available |
| 8 | 55 years | Female | Not available | Not available |

Notes. * indicated antiviral therapy for HBV as well
